# Sleep Medication Use by people with Cerebral Palsy: A Population Level Data Linkage Study.

**DOI:** 10.23889/ijpds.v7i3.1906

**Published:** 2022-08-25

**Authors:** Lisa Kent, Mary-Elaine McCavert, Karen McConnel, Oliver Perra, Aideen Maguire, Claire Kerr

**Affiliations:** 1 Office for National Statistics; 2 ADR Wales, Welsh Government; 3 ADR Wales, Swansea University; 4 Northern Ireland Statistics and Research Agency

## Objectives

To (1) compare proportions of the population dispensed sleep medication, and rate (dispensations/month) and amount (milligrams/month) of dispensed sleep medication, in individuals with and without cerebral palsy (CP); and (2) describe dispensation of sleep medication within CP and non-CP cohorts with respect to sociodemographic and clinical characteristics.

## Approach

Individuals aged 6 -36 years (aligning with those known to the Northern Ireland CP Register [NICPR]), registered with a general practitioner at 01-January-2018, were identified within the National Health Application and Infrastructure System. Sleep medications dispensed 01-January-2018 to 31-December-2019 were extracted from the Enhanced Prescribing Database. Analysis was limited to melatonin due to small counts in other medications. Routine healthcare data was sourced from the Honest Broker Service (HBS). NICPR clinical data (CP-type, Gross Motor Function Classification System (GMFCS), gestation and birthweight) were linked to routine healthcare data using the Health and Care Number by HBS. Descriptive statistics are presented.

## Results

Complete matching was achieved between NICPR and healthcare data using the HCN. Final cohorts consisted of 1,598 individuals with CP and 790,097 without CP. A greater proportion of those with CP were dispensed melatonin compared to those without CP (4.6% vs 1.0%). The CP cohort were also dispensed melatonin at a greater rate (median(IQR) CP 0.33(0.71) vs non-CP 0.25(0.54) dispensations/month) and in greater amounts (median(IQR) CP 30(74.7) vs non-CP 17.5(55.0) mg/month). Within the CP cohort, differences in melatonin dispensation were observed across sociodemographic groups (male 5.1% vs female 3.9%; children 8.2% vs young adults 2.2%; urban 6.5% vs rural 5.0%); deprived 5.1% vs affluent 4.2%). Clinical characteristics associated with greatest dispensation of melatonin were non-spastic CP (6.83%), GMFCS IV&V (5.29%), or extremely premature birth (6.85%).

## Conclusion

Individuals with CP, particularly children, are more likely to be dispensed sleep medications compared to the general population. Awareness of this disparity could encourage further research on assessment and management of sleep in CP and facilitate discussions between healthcare providers and families on underlying causes of sleep problems.

